# Supplementary material for: EDEM2 stably disulfide-bonded to TXNDC11 catalyzes the first mannose trimming step in mammalian glycoprotein ERAD
Source: eLife. 2020 Feb 17;9:e53455. doi: 10.7554/eLife.53455 (PMC7039678; doi:10.7554/eLife.53455)
Supplement: Supplementary file 1. [file elife-53455-supp1.docx]

**Supplementary File 1**

| Reagent type or resource | Designation | Source or reference | Identifier | Additional information |
| --- | --- | --- | --- | --- |
| Sequence- based reagent | 753Fw | This paper | Genomic PCR primer | CTACAGCGGTGTCAAGGTTCCAAGTAT |
| Sequence- based reagent | 1708Rv | This paper | Genomic PCR primer | AAGTGACAGGTCCACCCCTAGTGACTC |
| Sequence- based reagent | 730Fw | This paper | Genomic PCR primer | AGACTACAGCGGTGTCAAGGTTCCAAGTAT |
| Sequence- based reagent | 1831Rv | This paper | Genomic PCR primer | TTAGATCAATGAGTGCGGGAATTCTAAAAG |
| Sequence- based reagent | TgFw | This paper | Genomic PCR primer | AGTATGACAGGAAAAACTTCCATTTTAGTG |
| Sequence- based reagent | TgRv | This paper | Genomic PCR primer | TATTAGGTCTGAAGAGGAGTTTACGTCCAG |
| Sequence- based reagent | TXNDC 11-Fw | This paper | RT-PCR primer | GGAATTCGGTCGGAATGCGGAGGCCGCGGC |
| Sequence- based reagent | TXNDC 11-Rv | This paper | RT-PCR primer | TCGGTCGACTTAGTCTGTCCTGTTCTCCTT |
| Sequence- based reagent | GM130- Fw | This paper | RT-PCR primer | GAAGAATTCTATGTCGGAAGAAACCCGAC |
| Sequence- based reagent | GM130- Rv | This paper | RT-PCR primer | GAAGTCGACTTAGATGACAGTGATCCTTCACC |
| Sequence- based reagent | 778Fw | This paper | qRT-PCR primer | GGAGAGCCGGTCAGATATCG |
| Sequence- based reagent | 838Rv | This paper | qRT-PCR primer | CCACTTGCCAGTGAGCACAT |
| Sequence- based reagent | 1036Fw | This paper | qRT-PCR primer | CCAGTCCTTGGAGGCCTACTG |
| Sequence- based reagent | 1105Rv | This paper | qRT-PCR primer | GGTCCTCATGGCATTGTCAAT |
| Sequence- based reagent | XBP1-Fw | This paper | qRT-PCR primer | CTGCTGAGTCCGCATCAGGT |
| Sequence- based reagent | XBP1-Rv | This paper | qRT-PCR primer | GAGTCAATACCGCCAGAATCCA |
| Sequence- based reagent | GAPDH-Fw | This paper | qRT-PCR primer | ATTCCATGGCACCGTCAAG |
| Sequence- based reagent | GAPDH-Rv | This paper | qRT-PCR primer | GATCTCGCTCCTGGAAGATG |
